# Synthesis of Tilmicosin Nanostructured Lipid Carriers for Improved Oral Delivery in Broilers: Physiochemical Characterization and Cellular Permeation

**DOI:** 10.3390/molecules25020315

**Published:** 2020-01-13

**Authors:** Benazir Sahito, Qian Zhang, Haifeng Yang, Lin Peng, Xiuge Gao, Jam Kashif, Zain ul Aabdin, Shanxiang Jiang, Liping Wang, Dawei Guo

**Affiliations:** 1 Laboratory of Veterinary Pharmacology and Toxicology, College of Veterinary Medicine, Nanjing Agricultural University, 1 Weigang, Nanjing 210095, China; DRbena22@hotmail.com (B.S.); zq99999@yeah.net (Q.Z.); 2016207003@njau.edu.cn (L.P.); vetgao@njau.edu.cn (X.G.); jamkashifsahito@hotmail.com (J.K.); drzainsahito@hotmail.com (Z.u.A.); nauvy@sina.com (S.J.); wlp71@163.com (L.W.); 2 Department of Animal Pharmacy, Jiangsu Agri-Animal Husbandry Vocational College, Taizhou 225300, China; yhf8142@sina.com

**Keywords:** tilmicosin, nanostructured lipid carriers, oral bioavailability, broiler, cellular permeability, P-gp efflux

## Abstract

This study aimed to develop nanostructured lipid carriers (NLCs) for improved oral absorption of tilmicosin (TMS) in broilers. Thus, palmitic acid, lauric acid, and stearic acid were selected as solid lipids to formulate TMS-pNLCs, TMS-lNLCs, and TMS-sNLCs, respectively. They showed similar physicochemical properties and meanwhile possessed excellent storage and gastrointestinal stability. The TMS interacted with the lipid matrix and was encapsulated efficiently in NLCs in an amorphous structure. NLCs could enhance oral absorption of TMS compared to 10% tilmicosin phosphate solution in broilers, among which the TMS-sNLCs were the most efficient drug delivery carriers, with a relative oral bioavailability of 203.55%. NLCs could inhibit the efflux of P-glycoprotein (P-pg) toward TMS, which may be involved with improved oral absorption. Taken together, these types of solid lipids influenced the enhanced level of NLCs toward oral bioavailability of TMS, and the sNLCs proved to be the most promising oral delivery carriers of TMS.

## 1. Introduction

Tilmicosin, derived from tylosin, is a bacteriostatic broad-spectrum macrolide antibiotic that has a wide range of veterinary uses, including treatment of bacterial and *Mycoplasma* infections (such as pulmonary infection) through inhibiting the biosynthesis of microbial protein. It has also been recently used to treat mastitis [1,2]. Previously, tilmicosin was approved to treat *Pasteurella* infection in swine and bovines and also control *Mycoplasma* infection in chickens [3]. Because of its poor water-solubility, tilmicosin phosphate has been engineered and extensively applied in veterinary medicine in a soluble form. However, this form of tilmicosin displays decline in absorption, low oral bioavailability, and less potency [4]. Additionally, tilmicosin treatment exerted several side effects such as gastrointestinal disorders, liver damage, and even cardiotoxicity at high doses [5,6]. Thus, there is still an urgent need to develop a novel formulation for improving the oral bioavailability of tilmicosin, thus decreasing the usage amounts and toxicity in the veterinary clinic. 

On the other hand, lipid-based nanocarriers are considered as an effective means of oral delivery of poorly water-soluble and lipophilic drugs owing to their potential to enhance the solubility and improve oral bioavailability [7]. Recently, it has been reported that lipid-based nanocarriers including nanostructured lipid carriers (NLCs) and lipid-core nanocapsules (LNCs) could enhance in vitro antibacterial activity against *Escherichia coli* and *Staphylococcus aureus* (*S. aureus*) in comparison to the native tilmicosin [8]. In addition, solid lipid nanoparticles (SLNs) also could strengthen in vitro antibacterial activity of tilmicosin against *S. aureus*, and significantly enhanced the therapeutic efficacy of tilmicosin in a mouse mastitis model infected with *S. aureus* [9]. It is known that overdose or intravenous injection of tilmicosin can lead to serious acute cardiac toxicity [6]. Interestingly, SLNs could markedly decrease the toxicity of tilmicosin in Institute of Cancer Research (ICR) mice [10]. NLCs are the second generation of lipid nanoparticles containing blended lipids (liquid oil and solid fat). The addition of liquid lipids delays the formation of perfect lipid crystals, thus increasing the loading capacity of lipophilic drugs, and decreasing drug leakage during storage compared to the SLNs [11,12]. However, NLCs have been established to increase the oral bioavailability of several poorly water-soluble drugs [13]. More importantly, NLCs show a higher cellular uptake and permeability across the barrier when compared to SLNs [14]. Therefore, we proposed that NLCs would be a promising drug delivery system for improving the oral bioavailability of tilmicosin in chickens.

Plenty of studies have suggested that physicochemical characteristics of NLCs may influence the oral bioavailability of poorly water-soluble drugs. Smaller NLCs showed greater penetration, oral absorption, and bioavailability than larger particles [15,16]. The administration of positively charged lipid nanoparticles can promote the binding to mucosa with negative charges, and consequently increase their residence time [7]. NLCs are prepared using solid lipids, liquid lipids, and surfactant. The proportion for these components plays a vital role in defining the physicochemical characteristics of the resulting NLCs. In addition, the different chemical compositions also resulted in a difference in the cellular permeability of NLCs [17,18]. However, the effect of solid lipid types on the oral bioavailability of nanostructured lipid carriers for tilmicosin in broilers remains to be investigated.

In this study, we firstly prepared three types of tilmicosin-loaded nanostructured lipid carriers (TMS-NLCs) with different solid lipids via high shear together with the ultrasonic method, and then physiochemical properties including morphologies, hydrodynamic diameters, zeta potentials, stability, and interaction between drug and carriers were also characterized. Furthermore, we performed an in vivo pharmacokinetics study of three newly prepared formulations in comparison with the commercial tilmicosin phosphate formulation (10% TMS) via oral administration in broilers. Finally, Caco-2 cell monolayers were used to evaluate the in vitro absorption efficacy and interaction with P-glycoprotein (P-gp) efflux for these formulations. Taken together, the required data may reveal the effect of solid lipids on the physicochemical properties and oral bioavailability of TMS-NLCs in broilers.

## 2. Results and Discussion

### 2.1. Characterization of TMS-NLCs

**Figure 1:**
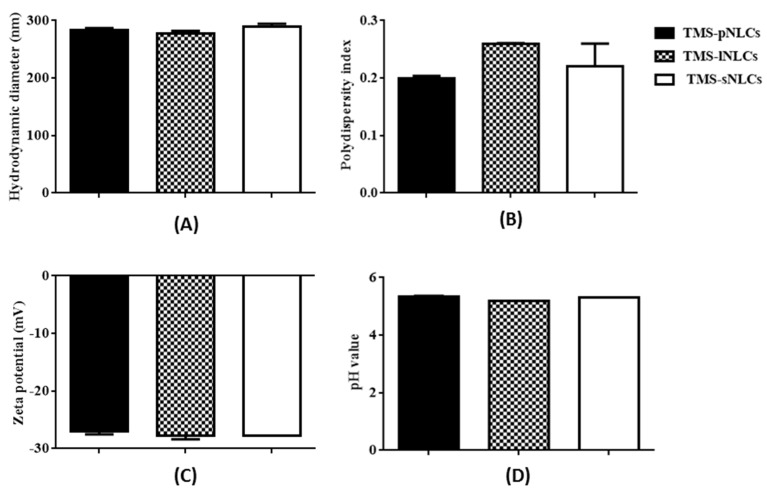
Hydrodynamic diameters (**A**), polydispersity index (**B**) and zeta potentials (**C**), and pH values (**D**) of tilmicosin-loaded nanostructured lipid carriers with palmitic acid (TMS-pNLCs), lauric acid (TMS-lNLCs), and stearic acid (TMS-sNLCs) in aqueous solution.

**Figure 2:**
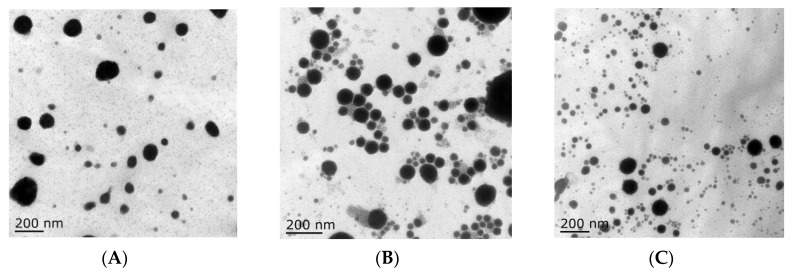
The morphology of TMS-pNLCs (**A**), TMS-lNLCs (**B**), and TMS-sNLCs (**C**) observed by transmission electron microscopy.

In this study, tilmicosin-loaded nanostructured lipid carriers (TMS-NLCs) were manufactured using high shear together with the ultrasonication method. In order to evaluate the effect of solid lipids on the intestinal absorption of tilmicosin, palmitic acid (PA), lauric acid (LA), and stearic acid (SA) were employed as solid lipids, and mixed with oleic acid to prepare the various types of NLCs, which were TMS-pNLCs, TMS-lNLCs, and TMS-sNLCs, respectively. Particle size is considered as the fundamental index for the recognition of nanoparticles and plays an important role in the evaluation of nanoparticles [19]. Therefore, the hydrodynamic diameters (HDs) of NLCs were firstly measured by dynamic light scattering via Zetasizer Nano ZS, and the results showed they had a similar diameter and narrow size distribution, at 283.5 ± 3.53 nm, 277.5 ± 4.52 nm, and 289.5 + 5.06 nm, respectively (Figure 1A). Earlier reports suggested that particles around 300 nm in size are ideal for oral drug delivery since they are preferentially up taken by both enterocytes and M cells [20]. The polydispersity index (PDI) values of all the formulations were less than 0.3 (Figure 1B), suggesting excellent monodispersity and homogeneity in the aqueous solution. The zeta potential is of importance to characterize the storage stability of nanomaterials [21]. Additionally, the static repulsion of the nanoparticles with the same surface charge can provide extra stability [22]. Here, the three types of TMS-NLCs displayed negative surface charges with zeta potential of more or less −27 mV (Figure 1C), indicating a good physical stability since strong electrostatic repulsion could hinder the aggregation between particles. Furthermore, the use of steric stabilizers has been also shown to produce stable formulations [23]. Moreover, two non-ionic emulsifiers (poloxamer-407 and Tween 20) were used to sterically stabilize the NLCs by forming a hydrophilic coat around their surface [24]. Additionally, it was obvious that the morphology of TMS-NLCs was in an approximately spherical shape and they were non-adherent to each other with a smooth surface under TEM (Figure 2). The size was smaller than the HD, as determined by dynamic light scattering (DLS). TMS-NLCs would be highly hydrated due to the adsorption of the oil in water emulsifiers Tween 20 and poloxamer-407 on the surface of mixed lipids. As reported earlier, HD measurements are performed by DLS in an aqueous state, implying that highly hydrated lipid nanoparticles exhibited larger diameters compared to non-hydrated ones [25]. All three kinds of TMS-NLCs were successfully prepared and exhibited similar physicochemical properties. 

**Figure 3:**
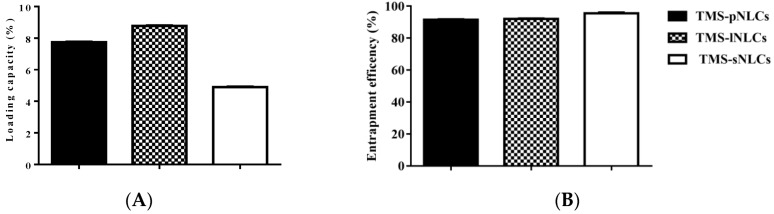
Entrapment efficiency (**A**) and loading capacity (**B**) of the newly prepared TMS-pNLCs, TMS-lNLCs, and TMS-sNLCs.

**Figure 4:**
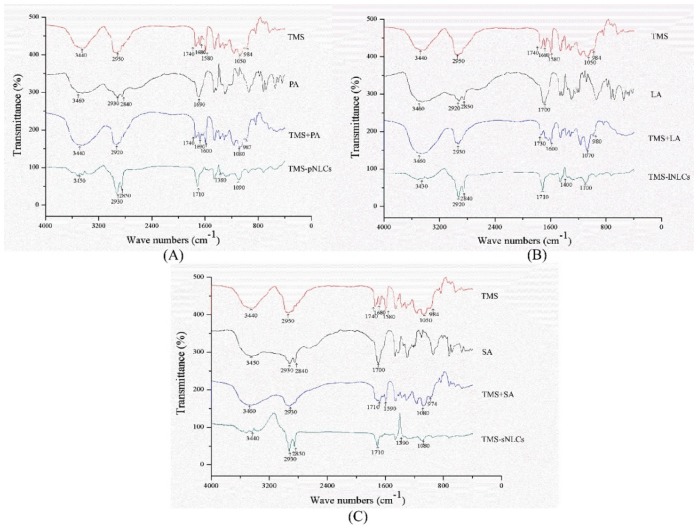
Fourier transformed infrared spectroscopy (FT-IR) of TMS-pNLCs (**A**), TMS-lNLCs (**B**), and TMS-sNLCs (**C**).

**Figure 5:**
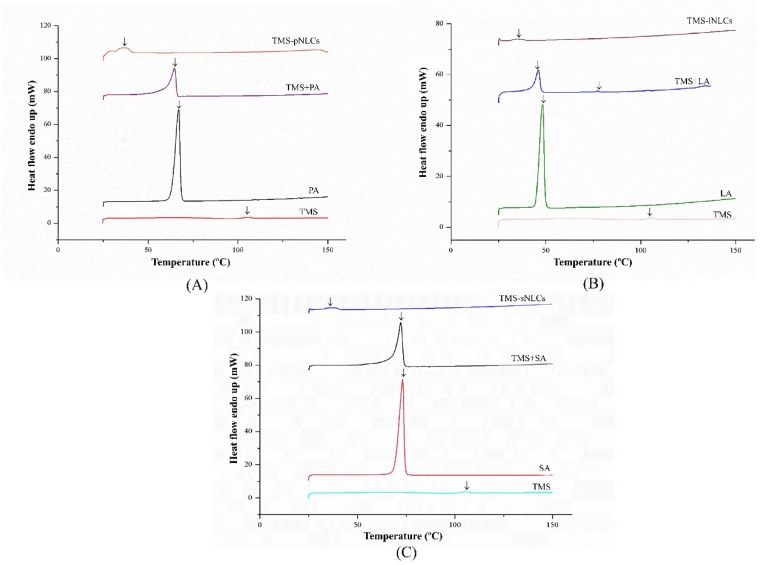
Differential scanning calorimetry (DSC) curves of TMS-pNLCs (**A**), TMS-lNLCs (**B**), and TMS-sNLCs (**C**).

Entrapment efficiency (EE) and loading capacity (LC) are two important factors to appraise the efficiency of carriers [26]. Loading capacity varied with the formulation change due to the solubility differences of TMS in solid lipids. Higher EE and LC values represent a more powerful ability of carriers. The solubility profile of poorly water-soluble drugs in a lipid matrix plays a key role in EE and LC of NLCs, and therefore the LC of NLCs varied with the change of lipid types [27]. In this study, due to the varieties in the solubilizing capacity of TMS in various solid lipids (Table S1), the LCs of as-prepared TMS-pNLCs, TMS-lNLCs, and TMS-sNLCs were 7.74 ± 0.03%, 8.78 ± 0.02%, and 4.9 ± 0.03%, respectively, while their corresponding encapsulation efficiency (EE) values were 91.47 ± 0.32%, 92.03 ± 0.24%, and 95.50 ± 0.53%, respectively (Figure 3). The high EE of TMS in the NLCs can suggest a less ordered arrangement in the lipid structure and increased imperfections that allow incorporation of drugs into the lipid matrix [28]. Fourier transformed infrared spectroscopy (FT-IR) is a valuable qualitative tool for characterizing the interaction of the components in pharmaceutical systems [29]. FT-IR analysis was further carried out for pure TMS, solid lipids, liquid lipids, and the TMS physical mixture as well as freeze-dried TMS-NLCs. As shown in Figure 4, the absorption peaks of pure TMS with O-H stretching vibration around at 3440 cm^−1^, C-H (methyl) antisymmetric stretching vibration around 2940 cm^−1^, C=O (inner ester) stretching vibration at 1740 cm^−1^ and 1680 cm^−1^, C=C (inner ester) stretching vibration at 1580 cm^−1^, C-O (inner ester or pyranyl) stretching vibration at 1050 cm^−1^, and C-H (pyranyl or piperidyl) flexural vibration at 984 cm^−1^ were observed. In terms of TMS-NLCs preparations, a characteristic absorption peak of TMS at 1580 cm^−1^ and 1680 cm^−1^ disappeared, and the C=O (inner ester) stretching vibration at 1740 cm^−1^ still existed, but its position was slightly altered (1710 cm^−1^ and 1640 cm^−1^, respectively). These results further demonstrated that TMS could be efficiently encapsulated in the NLCs by interacting with the lipid matrix and are consistent with a previous report that differential scanning calorimetry (DSC) analysis is frequently utilized to evaluate the melting behavior or crystallization [30]. Thermograms of pure TMS, solid lipids, solid lipids, and the TMS physical mixture, as well as TMS-NLCs preparations, were detected by DSC (Figure 5). The TMS had a weak endothermic peak at 106.12 °C. The DSC curves of PA, LA, and SA exhibited a flat profile with sharp endothermal peaks at 67.06 °C, 48.22 °C, and 74.18 °C, which were typical crystal structure characteristics. In contrast, the characteristic peak of all solid lipids in NLCs migrated, and even almost disappeared, while the endothermic peak of TMS completely disappeared in NLCs. These DSC data indicate that TMS existed in the carrier materials of the TMS-NLCs formulation in an amorphous or disordered structure [31].

### 2.2. The Stability of TMS-NLCs

#### 2.2.1. Storage Stability of TMS-NLCs

**Figure 6:**
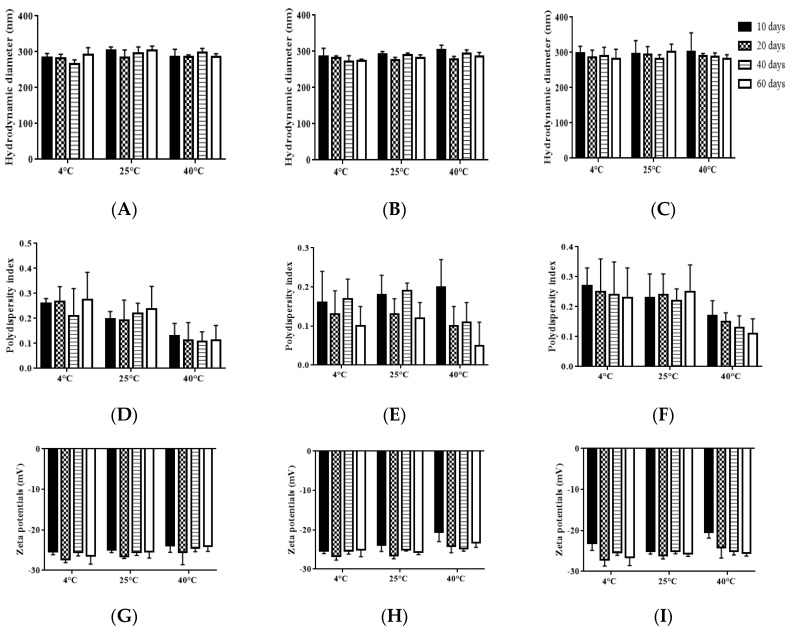
Storage stability of TMS-NLCs after 60 days of storage at 4 °C, 25 °C, and 40 °C. (**A**–**C**) Hydrodynamic diameters of TMS-pNLCs, TMS-lNLCs, and TMS-sNLCs, respectively. (**D**–**F**) The polydispersity index of TMS-pNLCs, TMS-lNLCs, and TMS-sNLCs. (**G**–**I**) The exhibited zeta potentials of TMS-pNLCs, TMS-lNLCs, and TMS-sNLCs, respectively. All the values are presented as means ± SD (*n* = 3).

**Figure 7:**
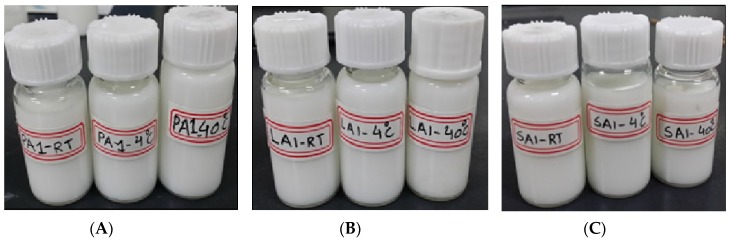
The physical appearance of TMS-pNLCs (**A**), TMS-lNLCs (**B**), and TMS-sNLCs (**C**) at 4 °C, 25 °C, and 40 °C after 60 days of storage.

Physical stability is considered as one of the most important features to ensure the safety and effectiveness of pharmaceutical preparations [27]. Firstly, the storage stability of each TMS-NLC suspension was investigated at 4 °C, 25 °C, and 40 °C for 60 days. The samples were taken out to determine their hydrodynamic diameters, polydispersity index, and zeta potentials at different storage times. The results showed there were no significant changes in these parameters during the whole storage period of each formulation (Figure 6). In addition, the physical appearances of TMS-pNLCs, TMS-lNLCs, and TMS-sNLCs were not evidently changed after 60 days of storage at 4 °C, 25 °C, and 40 °C (Figure 7). All these results convincingly indicated that the three TMS-NLCs preparations had excellent storage stability, which may be due to powerful steric hindrance and strong static repulsion between NLCs as mentioned above. 

#### 2.2.2. Stability of TMS-NLCs in the Stimulated Gastrointestinal Liquid

**Figure 8:**
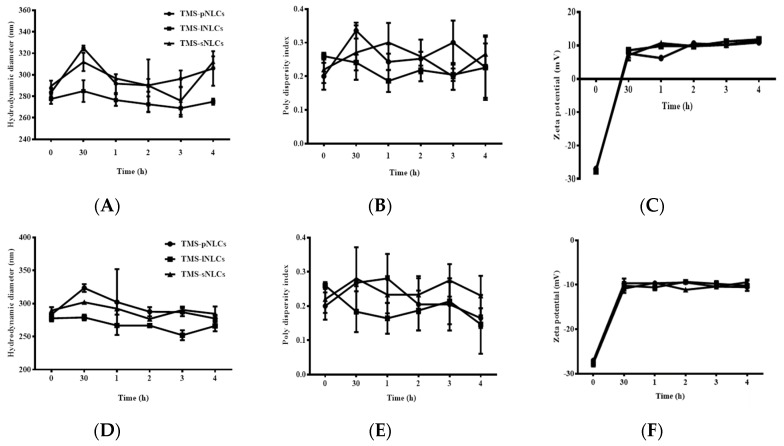
The stability of TMS-NLCs in the simulated gastric fluids (pH 1.23) and intestinal fluids (pH 6.8) during 4 h. (**A**–**C**) Hydrodynamic diameters, polydispersity index, and zeta potentials of TMS-pNLCs, TMS-lNLCs, and TMS-sNLCs in the simulated gastric fluids, respectively. (**D**–**F**) Hydrodynamic diameters, polydispersity index, and zeta potentials of TMS-pNLCs, TMS-lNLCs, and TMS-sNLCs in the simulated intestinal fluids, respectively.

As shown in Figure 1D, the pH values of the three TMS-NLCs solutions were in a narrow range from 5.185 to 5.335 without marked differences. It is well-known that the harsh gastrointestinal (GI) environment is the main hindrance influencing the stability of nanoparticles as drug oral delivery carriers [32]. TMS-NLCs as an efficient oral formulation should not be destroyed before reaching the absorption sites, and thus the stability of TMS-NLCs was studied in the simulated gastric liquid with pepsin (pH 1.2) and intestinal liquid with trypsin (pH 6.8), respectively. As shown in Figure 8C,F, zeta potentials of TMS-NLCs varied with pH change in the simulated GI liquid, and especially in acidic pH environment they were reversed from anions to cations, but no longer changed over time. There were no significant changes in the hydrodynamic diameters for any of the formulations in the stimulated GI liquid (Figure 8A,D). Similarly, the PDI values were less than 0.2 in the simulated GI liquid (Figure 8B,E). To sum up, these data clearly suggested that the TMS-NLCs could exist stably in the simulated GI fluids without being destroyed.

### 2.3. In Vivo Pharmacokinetic Study

**Figure 9:**
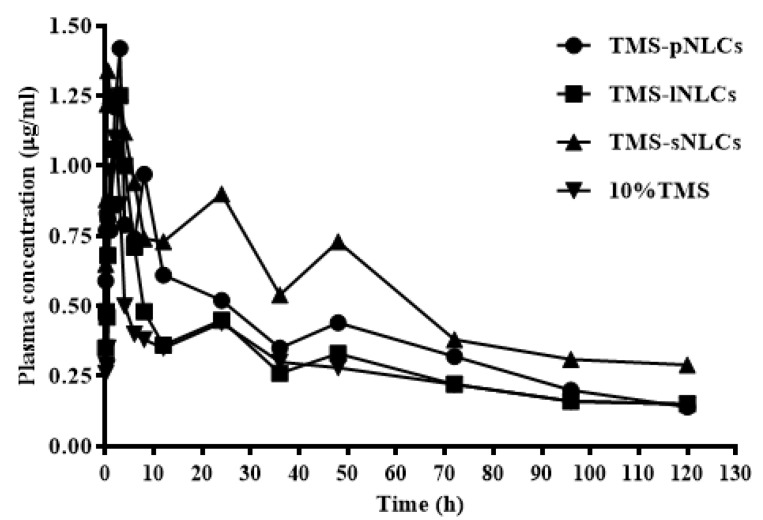
The drug concentration-time curves of TMS after a single oral administration of TMS-NLCs and 10% TMS with 25 mg/kg body weight (B.W.) in broilers (*n* = 10).

Several studies have indicated that the NLC formulation has a promising perspective to improve the oral bioavailability of poorly soluble drugs [33,34]. In the current study, plasma concentration time profiles and the corresponding pharmacokinetic parameters after oral administration of 10% TMS, TMS-pNLCs, TMS-lNLCs, and TMS-sNLCs in broilers at a single TMS dose of 25 mg/kg are presented in Figure 9 and Table 1, respectively. The pharmacokinetic behaviors of TMS after oral administration of 10% TMS and TMS-NLCs were fitted to the non-compartment model. As shown in Table 1, the time to reach the maximum amount (T_max_) of 10% TMS was 1.85 ± 0.67 h, while that of TMS-pNLCs, TMS-lNLCs, and TMS-sNLCs was delayed to 3.23 ± 1.99 h, 2.85 ± 1.53 h, and 2.12 ± 1.32 h, respectively. Meanwhile, the maximum concentration (C_max_) of the three preparations was slightly increased from 1.45 ± 0.88 μg/mL to 1.89 ± 0.60 μg/mL, 1.51 ± 0.32 μg/mL, and 1.97 ± 0.81 μg/mL, respectively. In addition, the areas under the curve from zero to last time (AUC_0-t_) of 10% TMS, TMS-pNLCs, TMS-lNLCs, and TMS-sNLCs were 32.42 ± 12.86 μg·h/mL, 44.75 ± 17.75 μg·h/mL, 35.23 ± 5.39 μg·h/mL, and 65.99 ± 18.98 μg·h/mL, respectively, suggesting all NLC formulations could improve the relative oral bioavailability of TMS, among which TMS-sNLCs had the highest relative oral bioavailability (203.55%) in comparison with 10% TMS. Interestingly, the mean residence time from zero to last time (MRT_0–t_) of TMS-sNLCs formulations were slightly changed compared to 10% TMS, indicating the withdrawal time of tilmicosin should be appropriately delayed when used in avian disease therapy. Overall, the TMS-sNLCs were the most efficient drug delivery carrier of TMS orally administrated in broilers. 

### 2.4. Evaluation of TMS Permeability across Caco-2 Cell Monolayers

In order to elucidate the mechanism of TMS-NLCs enhancing the oral absorption of TMS, the effect of NLCs on the TMS permeability across Caco-2 cell monolayers was investigated. In our previous study, tilmicosin was proved to be a substrate of P-gp, a drug efflux transporter [35]. Herein, we found that the efflux rate of 10% TMS in Caco-2 cell monolayers was 2.29 (Table 2), far higher than the 1.5 regulated by the United States Food and Drug Administration [36], further demonstrating that TMS as the substrate of P-gp could be removed from intestinal epithelial cells and its oral absorption thereby decreased [25]. In order to maintain the integrity of Caco-2 cell monolayers, the effect of TMS-NLCs on the viability of Caco-2 cells was firstly investigated to acquire the appropriate treatment dose. The results showed that all the TMS-NLC preparations could inhibit the proliferation of Caco-2 cells in a dose-dependent manner (Figure S1). In the present study, TMS-pNLCs, TMS-lNLCs, and TMS-sNLCs at the concentration of 10 μg/mL were selected to further evaluate TMS permeability across Caco-2 cells monolayers. The data displayed that the efflux rates of TMS-pNLCs, TMS-lNLCs, and TMS-sNLCs were reduced to 1.62, 1.56, and 1.88 (Table 2), respectively, suggesting that NLCs could reduce the efflux of P-gp against TMS, and thus could improve the oral bioavailability of TMS in chickens. The inhibition of TMS-pNLCs, TMS-lNLCs and TMS-sNLCs toward P-gp efflux is in correspondence with the concept that the nonionic surfactants such as Tween and poloxamer could alter the function of carriers/transporters. Additionally, the amounts of surfactants that inhibit the efflux transporters such as P-gp, breast cancer resistance protein (BCRP), and multidrug resistance protein (MRP2) are comparable in cell cultures [37]. However, the reduced levels of them were not in line with the improved levels of their corresponding oral relative bioavailability. Thus, inhibiting the P-gp efflux was not the only reason the NLCs heightened the oral bioavailability of TMS in broilers. The relevant details remain to be clarified in a follow-up study.

## 3. Materials and Methods

### 3.1. Materials

Tilmicosin (TMS, 91.4%) was purchased from Ningxia Teirui Pharmaceutical Co., Ltd. (Yinchuan, China). Tilmicosin standard (93.9%) was supplied by China Institute of Veterinary Drug Control (Beijing, China). Palmitic acid, oleic acid, and lauric acid were purchased from Aladdin Industrial Corporation (Shanghai, China). Stearic acid was obtained from Sinopharm Group Chemical Reagent Co., Ltd (Shanghai, China). Tween 20 was purchased from Nanjing Chemical Reagent Co., Ltd (Nanjing, China). Poloxamer-407 was obtained from Shanghai Macrochemical Preparation Auxiliary Technology Co. Ltd. (Shanghai, China). Phosphotungstic acid negative staining solution (5%) was purchased from Shanghai Source Leaf Biological Technology Co., Ltd. Methanol, acetonitrile, and tetrahydrofuran (HPLC grade) were obtained Honeywell Trade (Shanghai) Co., Ltd. Chloroform (analysis pure) was bought from Shanghai Ling Feng Chemical Reagents Co., Ltd. All other chemicals and reagents used were of analytical grade.

### 3.2. Preparation of Tilmicosin-Loaded Nanostructured Lipid Carriers

Tilmicosin-loaded nanostructured lipid carriers (TMS-NLCs) were fabricated using high-shear followed by an ultrasonication process according to our previous report, with some minor modifications [19]. Briefly, the specific amounts of TMS, Tween 20, and oleic acid were blended with melted palmitic acid, lauric acid, or stearic acid, respectively, to acquire an oil phase under stirring at 12,000 rpm at 70 °C. In addition, poloxamer-407 was dissolved in the double-distilled water at the same temperature to obtain the aqueous phase. Subsequently, the oil phase was immediately poured into the aqueous phase when they were heated; then the oil-in-water pre-emulsion was manufactured using aHigh Shear Dispersion Emulsifying Machine (FM200, IKA, Germany) at 11,000 rpm for 5 min, followed by ultrasonication at 300 W amplitude for 10 min with a Probe Ultrasonic Cell Disruptor (JY96-II, Scientz, Ningbo, China). The resulting hot emulsion was immediately transferred into the same volume of cold water at 4 °C for lipid solidification, and meanwhile dispersed by high shear of 11,000 rpm for 1 min to prepare the particle suspension. 

### 3.3. Measurement of Hydrodynamic Diameter and Zeta Potential of TMS-NLCs

The hydrodynamic diameter, polydispersity index (PDI), and zeta potential of the as-prepared formulations were measured by dynamic light scattering via Zetasizer Nano ZS (Nano-ZS90, Malvern Instruments, Worcestershire, UK) equipped with a back scattered light detector operating at 90° and equilibration time of 120 s. All the samples were diluted ten-fold with double-distilled water prior to measurement. All the measurements were performed in triplicates at 25 °C.

### 3.4. Determination of Entrapment Efficiency and Drug Loading of TMS-NLCs

Centrifugal ultrafiltration method was established to measure entrapment efficiency (EE) and loading capacity (LC). TMS-NLCs were transferred into a centrifugal ultrafiltration tube with molecular-weight cut off (MWCO) of 100 kDa and centrifuged at 5000 rpm for 20 min to separate free drug from TMS-NLCs. In addition, the TMS-NLC samples were added with adequate amounts of methanol, vortexed for 2 min, sonicated for 10 min, and centrifuged at 5000 rpm for 10 min. The collected supernatant was taken with a 0.22-μm syringe filter, and then 20 μL of filtrate were detected by high-performance liquid chromatography (HPLC). The EE and LC were calculated according to the following formula:

EE (%) = (W_total_ − W_free_)/W_total_ × 100%

LC (%) = (W_total_ − W_free_)/W_fat_ × 100%

where W_total_, W_free_, and W_fat_ are the total amounts of TMS, the unincorporated TMS, and the amounts of lipids and emulsifiers in the formulation, respectively. 

### 3.5. The Particle Morphology of TMS-NLCs

The morphological characteristics of TMS-NLCs were observed by transmission electron microscopy (TEM, Tecnai 12, Philips, Amsterdam, The Netherlands). Prior to the analysis, a drop of TMS-NLCs dispersion was spread on a 200 mesh copper grid coated with carbon membranes, and extra droplets were immediately removed. Phosphotungstic acid (2%, w/w) was dropped on the grid to negatively stain the particle. The negative staining samples were then dried at room temperature before the TEM analysis.

### 3.6. Fourier Transformed Infrared Spectroscopy of TMS-NLCs

Prior to the lyophilization process, TMS-NLCs were frozen overnight at −80 °C. An Alpha 2–4 LD plus a freeze-dryer (Martin Christ GmbH, Osterode, Germany) was used for freeze-drying and the operation circumstances were set at −60 °C and 0.011 mbar pressure. An FT-IR spectrophotometer (IR-470; Shimadzu, Kyoto, Japan) was used to record the FT-IR spectra of the selected formulations (TMS-pNLCs, TMS-lNLCs, and TMS-sNLCs). Correspondingly, solid components and physical mixtures were noted. The samples in the powder form were mixed with potassium bromide in a ratio of 100:1 and hard-pressed into pellet by high pressure hydraulic machine to conduct FT-IR.

### 3.7. Differential Scanning Calorimetry of TMS-NLCs

The thermal behaviors of the selected formulations (TMS-pNLCs, TMS-lNLCs, and TMS-sNLCs), the individual solid components, and physical mixtures were examined through differential scanning calorimetry (DSC 60; Shimadzu Corporation, Tokyo, Japan) on lyophilized samples. Each sample was wrapped in aluminum pans and heated at a temperature range of 40~200 °C with 10 °C/min at constant rates. The TA 50I PC system with (Shimadzu software programs) was used to record the thermal analysis data. The DSC temperature and enthalpy scale were rectified through the Indium standard.

### 3.8. Stability Analysis of TMS-NLCs

The stability was performed for the three formulations, including TMS-pNLCs, TMS-lNLCs, and TMS-sNLCs. All as-prepared formulations were stored at 4 °C, 25 °C, and 40 °C for 60 days. Samples were withdrawn at fixed time intervals and the formulations were evaluated for physical appearance, particle size, polydispersity index, and zeta potential. Additionally, the particle characteristics were examined after mixing with the buffers of different pH values (pH 1.2 and pH 6.8) to reveal the stability of TMS-NLCs in the gastrointestinal environment.

### 3.9. In Vivo Examination

All animal experiments were performed in accordance as approved by the Institutional Animal Care and Use Committee at the Nanjing Agricultural University (IACUC20170030101). An in vivo examination was performed to compare the oral bioavailability of three newly prepared TMS-NLCs with the marketed formulation (10% TMS) in broilers after a single dose of TMS by oral administration. 

#### 3.9.1. Induction of Experiment

Forty healthy broiler chickens at the age of 30 days with average body weight 1.2 to 1.4 kg were selected for this study. The chickens were caged at dark/light cycle of 12 h with the temperature of 25 ± 2 °C and humidity of 55 ± 10%. Feed and fresh water were available to them throughout the whole period. Monitoring of the chickens was done up to 7 days of adaptation period before the administration of the drugs. Chickens were off feed 12 h prior to drug administration and 6 h after drug dosing. However, they had free availability of water. 

#### 3.9.2. Pharmacokinetic Study

This experiment was done to explore the capability of NLCs to enhance the oral bioavailability of TMS. The experimental chickens were randomly separated into four groups (*n* = 10). All the formulations were freshly prepared. TMS-pNLCs, TMS-lNLCs, TMS-sNLCs, and 10% TMS were administered orally using a single dose of 25 mg/kg TMS. Blood samples (0.5 mL) were obtained via wing vein at designated time points (0, 5 min, 10 min, 20 min, 30 min, 1 h, 2 h, 3 h, 4 h, 6 h, 8 h, 12 h, 24 h, 36 h, 48 h, 72 h, and 96 h) after oral administration of a single dose, and were transferred into anticoagulant tubes containing heparin. The plasma was isolated through centrifugation at 3000 rpm for 10 min. The plasma samples were stored at −20 °C refrigerator for further analysis. 

Then, 20 μL of plasma samples were mixed with methyl alcohol (280 µL), and centrifuged at 5000 rpm (Eppendorf centrifuge 5424R) for 10 min at 4 °C. The special glass tube was used to transfer supernatant and 20 μL of each sample was injected for the HPLC detection. The HPLC method was executed through a series of Agilent technologies 1200. The HPLC column was an Agilent Zorbax Eclipse XDB-C18 (4.6 × 250 mm, 5 μm). The HPLC examination was done through a mobile phase, which comprises acetonitrile, tetrahydrofuran, phosphate butylamine buffer, deionized water (adjusted pH to 2.5 by phosphoric acid) at 12:5.5:2.5:80 (*v*/*v*/*v*/*v*). A 0.45-μm membrane filter was used for mobile phase filtration under the vacuum. For chromatography, a parting 1 mL/min flow rate, 20 μL injection volume, and DAD detector were used to analyze elute at a wavelength of 291 nm. 

#### 3.9.3. Pharmacokinetic Analysis

The software 3P97 was used to calculate the pharmacokinetic parameters matching the plasma concentration-time data to a suitable model compiled by (The Chinese Academy of Pharmacology Mathematics Specific Committee). TMS concentrations in plasma for each bird were used to determine their concentration–time profile. The Marquardt method was used to determine the area under the curve from zero to last time (AUC_0-t_). The maximum concentration (C_max_), time to reach the maximum amount (T_max_), and mean residence time (MRT) were analyzed. In addition, the relative oral bioavailability (F) of three formulations was calculated using AUC_0-t_ in comparison with 10%TMS.

### 3.10. In Vitro Studies for Cellular Permeation and Transport Activity Assay

Caco-2 cells were cultured at 37 °C under a 5% CO2 atmosphere in Dulbecco Modified Eagle Medium (DMEM) supplied with 10% fetal bovine serum (FBS) and 1% penicillin–streptomycin mixture. Caco-2 cells were sowed with 2 × 10^5^ cells per transwell^®^ insert (12-well, 0.4 μm pore diameter, Corning Costar, Cambridge, MA, USA) to acquire cell monolayers. DMEM at 0.5 mL and 1.5 mL were put in the apical side (AP) and basolateral side (BL) of the transwell^®^ insert, respectively. The transepithelial electrical resistance (TEER) was determined by the Epithelial Volt-Ohm Meter (Millicell^®^ ERS-2, Millipore, Billerica, MA, USA) to authenticate the integrity of the cell monolayers. After 21 days of culturing, cell monolayers had TEER values of more than 450 Ω cm^2^ and were utilized to investigate the transport of TMS-NLCs. In addition, the TEER values of transcellular models were monitored during the transport process of TMS-NLCs to guarantee the integrity of cell monolayers.

The permeability of TMS across Caco-2 cell monolayers was investigated by comparing 10% TMS with TMS-sNLCs. The bidirectional transport assay was carried out at 37 °C. The samples were collected from the BL or AP side after 4 h of incubation, and the concentrations of TMS were detected by HPLC. The apparent permeability coefficients (P_app_, cm/s) for both AP→BL (P_app_(AP→BL)) and BL→AP (P_app_(BL→AP)) were calculated as Formula (1), where dQ/dt, A, and C_0_ were the transport rate, the surface area of the filter membrane, and the initial concentration of the tested drug, respectively. The efflux ratio (ER) value was obtained from the ratio of P_app_ (BL→AP) to P_app_ (AP→BL) using Formula (2).

(1) $$P_{\mathrm{app}}=\left(\mathrm{dQ}/\mathrm{dt}\right)/\left({A\times C}_{0}\right)$$

(2) $$ER=\frac{P_{\mathrm{app}}\left(\mathrm{BL}\to \mathrm{AP}\right)}{P_{\mathrm{app}}\left(\mathrm{AP}\to \mathrm{BL}\right)}$$

### 3.11. Statistical Analysis

The results were expressed as mean values ± standard deviation (SD). Statistical analysis was performed using the one-way analysis of variance (ANOVA) on SPASS 23 (SPASS Inc., Chicago, IL, USA). Differences were considered significant at * *p* < 0.05. The graphs were plotted by using GraphPad Prism 7 (GraphPad InStat Software, USA). 

## 4. Conclusions

Conclusively, three TMS-NLC formulations were successfully prepared and possessed similar physiochemical properties. However, TMS-pNLCs, TMS-lNLCs, and TMS-sNLCs displayed different pharmacokinetic behaviors when administrated orally at the dose of 25 mg/kg in broilers, among which TMS-sNLCs had the highest relative oral bioavailability compared to the marketed formulation (10% TMS). The transport across the Caco-2 cell monolayers further revealed that inhibition of NLC against P-gp efflux was involved in enhanced oral absorption of TMS. Nevertheless, the inhibition of TMS-sNLCs against the efflux of P-gp was not the strongest amongst the three TMS-NLCs formulations, which was not inconsistent with the enhanced degrees of various TMS-NLC formulations toward oral bioavailability in broilers. Taken together, the solid lipid composition of NLCs influenced the oral absorption of TMS, and the TMS-sNLC formulation was one of the most efficient oral delivery carriers of TMS in broilers.

## Figures and Tables

**Table 1:** Pharmacokinetic parameters of 10%TMS, TMS-pNLCs, TMS-lNLCs, and TMS-sNLCs orally administrated in the broiler chickens (non-compartmental model). C_max_: maximum concentration; T_max_: time to reach the maximum amount; AUC_0–t_: area under the curve from zero to last time; MRT_0-t_: mean residence time; F: relative oral bioavailability.

Parameters	10% TMS	TMS-pNLCs	TMS-lNLCs	TMS-sNLCs
T_max_ (h)	1.85 ± 0.67	3.23 ± 1.99 *	2.85 ± 1.53	2.12 ± 1.32
C_max_ (μg/mL)	1.45 ± 0.88	1.89 ± 0.60	1.51 ± 0.32	1.97 ± 0.81 *
AUC_0__-–__-t_ (μg·h/mL)	32.42 ± 12.86	44.75 ± 17.75	35.23 ± 5.39	65.99 ± 18.98 *
MRT_0__-–__t_ (h)	43.82 ± 4.19	39.53 ± 4.76	42.04 ± 4.90	45.26 ± 4.80 *
F (%)	/	138.03	108.67	203.55

Note: * *p* < 0.05.

**Table 2:** Apparent permeability (P_app_) and efflux rate (ER) of 10% TMS, TMS-pNLCs, TMS-lNLCs, and TMS-sNLCs across Caco-2 cell monolayers. AP: apical side (AP); BL: basolateral side.

Formulations	P_app_ (×10^−6^ cm/s)		Efflux Rate (ER)
	AP→BL	BL→AP	
10% TMS	0.32 ± 0.08	0.74 ± 0.07	2.29
TMS-pNLCs	0.54 ± 0.06	0.88 ± 0.05	1.62
TMS-lNLCs	0.65 ± 0.10	1.01 ± 0.07	1.56
TMS-sNLCs	0.57 ± 0.05	1.07 ± 0.13	1.88

## Supplementary Materials

The following are available online. Figure S1: Effect of 10% TMS, TMS-pNLCs, TMS-lNLCs, and TMS-sNLCs on the viability of Caco-2 cells. Table S1. The solubility of tilmicosin in different lipids.
