# Supplementary material for: Synthesis of Tilmicosin Nanostructured Lipid Carriers for Improved Oral Delivery in Broilers: Physiochemical Characterization and Cellular Permeation
Source: Molecules. 2020 Jan 13;25(2):315. doi: 10.3390/molecules25020315 (PMC7024240; doi:10.3390/molecules25020315)
Supplement: Supplementary file 1 [file molecules-25-00315-s001.pdf]

Supplementary materials

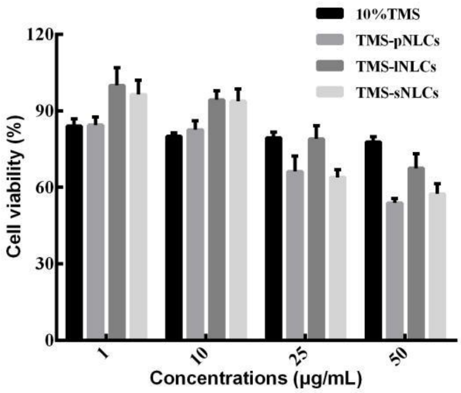

Figure S1. Effect of 10%TMS, TMS-pNLCs, TMS-lNLCs and TMS-sNLCs on the viability of Caco-2 cells.

Table S1. The solubility of tilmicosin in different lipids

Lipid type	Solubility of tilmicosin (g/g)
Plamitc acid	0.6
Lauric acid	0.5
Stearic acid	0.4
Oleic acid	0.6
